# Risk factors for drug-related cachexia: an analysis based on the FDA adverse event reporting system (FAERS) from 2004 to 2025

**DOI:** 10.3389/fphar.2026.1913539

**Published:** 2026-09-08

**Authors:** Lingli Huang, Shuchen Dong, Quanli Zhang, Jie Ni

**Affiliations:** 1 Department of Pharmacy, Jiangsu Cancer Hospital, Jiangsu Institute of Cancer Research, The Affiliated Cancer Hospital of Nanjing Medical University, Nanjing, Jiangsu, China; 2 Department of Oncology, Jiangsu Cancer Hospital, Jiangsu Institute of Cancer Research, The Affiliated Cancer Hospital of Nanjing Medical University, Nanjing, Jiangsu, China; 3 Department of Scientific Research, Jiangsu Key Laboratory of Innovative Cancer Diagnosis & Therapeutics, Jiangsu Cancer Hospital, Jiangsu Institute of Cancer Research, The Affiliated Cancer Hospital of Nanjing Medical University, Nanjing, China

**Keywords:** cachexia, disproportionality analysis, FAERS, pharmacovigilance, risk factors

## Abstract

**Background:**

Cachexia is a devastating metabolic syndrome that has a profound impact on survival rate and quality of life. Currently, there is still a scarcity of systematic evidence regarding drug-induced cachexia. This study aims to identify drugs associated with cachexia through the first large-scale pharmacovigilance analysis, and to characterize their risk features using real-world data.

**Methods:**

Data from the U.S. FDA Adverse Event Reporting System (FAERS) from January 2004 and March 2025 were analyzed. Reports listing cachexia as an adverse event were extracted. Disproportionality analysis was performed on the top 50 most frequently reported drugs using reporting odds ratio (ROR), proportional reporting ratio, Bayesian Confidence Propagation Neural Network, and Multi-item Gamma Poisson Shrinker. A drug was considered a positive signal only if it met the threshold across all four methods. The Weibull shape parameter test was used to analyze the time-to-onset (TTO) profile.

**Results:**

Among 4,878 individual case safety reports of drug-related cachexia, the mortality rate was 25.6%. Disproportionality analysis identified 25 drugs with consistent positive signals across all four algorithms. These drugs spanned nine categories, with antineoplastic and immunomodulating agents being the most common. The drugs with stronger signals were low calcium peritoneal dialysis solution lactate G15 (ROR 495.67, 95% CI 336.94–729.18), Kaletra (ROR 10.44, 95% CI 7.98–13.65), and nivolumab (ROR 15.29, 95% CI 13.65–17.13). The drugs with a higher number of reported cases were nivolumab (319 cases), paclitaxel (160 cases), and Zometa (131 cases). The median TTO was 31 days, and more than half of cases occurred within the first 120 days, presenting an “early failure” pattern.

**Conclusion:**

This study identified 25 drug safety warning signals that are significantly associated with the occurrence of cachexia. Cachexia is more likely to occur in the initial stage of drug treatment and has a high mortality rate. Continuous regulatory review and label updates of the safety information of relevant medications should be promoted.

## 1 Introduction

Cachexia is a devastating metabolic syndrome characterized by progressive and unintentional loss of skeletal muscle mass, with or without fat loss, that cannot be fully reversed by conventional nutritional support. This complex multifactorial syndrome has been recognized throughout medical history, with descriptions dating back to ancient Greece when Hippocrates observed “the flesh is consumed and becomes water … the shoulders, clavicles, chest and thighs melt away.” The modern consensus definition, established through international efforts in 2011, emphasizes the persistent nature of skeletal muscle loss that leads to progressive functional impairment, distinguishing it from simple starvation, age-related muscle loss, or malabsorption (Yao et al., 2025; von Haehling and Anker, 2014). The pathophysiology of cachexia involves intricate interactions between pro-inflammatory cytokines, neurohormonal dysregulation, and metabolic alterations that create a perfect storm of catabolic processes, anorexia, and energy wasting (Yeom and Yu, 2022; Argiles et al., 2014; Carrero et al., 2013).

The clinical impact of cachexia extends far beyond weight loss, representing a systemic disorder that affects multiple organ systems and profoundly diminishes quality of life, treatment tolerance, and overall survival. In cancer patients, the prevalence of cachexia ranges from 50% to 80% in advanced stages, accounting for approximately 20% of cancer-related deaths (Koh et al., 2025; Meregaglia et al., 2019). Due to the increase in hospitalization rates, prolonged hospital stays, and heightened demands for supportive care, the medical expenses for patients with cachexia are 30%–40% higher, and the economic burden is equally heavy (Tarricone et al., 2016). Beyond oncology, cachexia manifests in chronic heart failure, chronic kidney disease, chronic obstructive pulmonary diseases, rheumatoid arthritis, and chronic infections such as HIV and tuberculosis, making it a truly cross-cutting challenge in modern medicine.

The molecular mechanisms driving cachexia are multifaceted, involving tumor-derived factors, systemic inflammation, and disrupted energy homeostasis. Pro-inflammatory cytokines including tumor necrosis factor-alpha (TNF-α), interleukin-1 (IL-1), and interleukin-6 (IL-6) activate ubiquitin-proteasome and autophagy-lysosome pathways, accelerating muscle protein degradation while simultaneously inhibiting protein synthesis (Rohm et al., 2019; Lin et al., 2025; Yeom and Yu, 2022). Concurrently, mitochondrial dysfunction and altered fatty acid metabolism contribute to inefficient energy production and utilization. The discovery that adipose triglyceride lipase-mediated lipolysis is promoted by TNF-α through depletion of the inhibitory protein G0S2 revealed specific molecular pathways connecting inflammation to tissue wasting (Zhao et al., 2025; Yang et al., 2011). These metabolic disturbances manifest clinically through anorexia, significant weight loss (>5% within 6 months), muscle wasting, fatigue, and functional impairment.

While cachexia has traditionally been viewed primarily as a complication of underlying diseases, emerging evidence suggests that pharmacological interventions may significantly contribute to or exacerbate this condition. Numerous therapeutic agents, while potentially life-prolonging, are associated with adverse effects that can directly precipitate or worsen cachexia. Chemotherapeutic agents such as platinum compounds and anthracyclines frequently cause gastrointestinal toxicities—nausea, vomiting, mucosal damage—that impair nutrient intake and absorption (Bozzetti, 2020). Innovative immunotherapies like ICIs (e.g., nivolumab) can induce immune-related adverse events mimicking chronic inflammatory states, potentially fueling the catabolic processes underlying cachexia (Masuda et al., 2019). Beyond oncology, antiretroviral therapies for HIV (e.g., lopinavir/ritonavir) contribute to mitochondrial toxicity and metabolic disturbances that promote muscle wasting (Margolis et al., 2014). Dopaminergic medications for Parkinson’s disease (e.g., levodopa) may induce nausea, vomiting, and appetite suppression, while peritoneal dialysis solutions potentially exacerbate protein-energy wasting in renal failure patients (Yeom and Yu, 2022; Groarke et al., 2024).

Despite these clinical observations, comprehensive studies systematically evaluating drug-induced cachexia remain remarkably limited. Most existing literature consists of isolated case reports or is embedded within studies focused primarily on the cachexia driven by the primary disease. The absence of large-scale, systematic pharmacovigilance studies specifically investigating drug-related cachexia risk across multiple therapeutic classes represents a significant knowledge gap in clinical medicine and drug safety.

The U.S. Food and Drug Administration (FDA) Adverse Event Reporting System (FAERS), containing over 10 million records of adverse drug events (ADEs), offers an invaluable resource for identifying potential drug-risk associations through disproportionality analysis techniques (Zorych et al., 2013). Previous pharmacovigilance studies have successfully utilized FAERS to detect ADEs associated with immunotherapies, antipsychotics, and antidepressants (Tan et al., 2025; Zhang et al., 2024; Kandukuru et al., 2024), but no similar comprehensive analysis has been conducted for drug-related cachexia. This study aims to comprehensively analyze the FAERS data, identify drugs associated with cachexia, describe the characteristics of affected patient populations and temporal patterns of occurrence, employ multiple disproportionality analysis methods to quantify risk signals, and identify risk factors. Our findings will enhance clinical recognition of drug-induced cachexia, facilitate risk stratification, inform monitoring strategies for vulnerable populations, and ultimately contribute to improved patient outcomes by enabling earlier intervention in this devastating syndrome.

## 2 Materials and methods

### 2.1 Data source and extraction

The data for this study were sourced from FAERS database, with the time period covering January 2004 and March 2025. The FAERS database is a database designed to support FDA’s post-market safety monitoring of drugs and therapeutic biologics and includes all ADE information and medication error information collected by the FDA. The FAERS data files contain seven types of data, including demographic and administrative information (DEMO), reaction information (REAC), drug information (DRUG), preferred terms (PTs), patient outcomes (OUTC), report sources (RPSR), therapy start and end dates for reported drugs (THER), indications for drug use (INDI).

Firstly, the data structure was standardized by unifying column names and merging quarterly data. Then, the variable values are standardized and the ADE terms were standardized using the system organ classifications (SOC) and preferred term (PT) from the International Medical Terminology Dictionary (MedDRA; version 26.1). The data was cleaned using the CASEID, FDA_DT and PRIMARYID in the DEMO table by the recommended method of FDA. The latest record of FDA_DT was retained for reports with the same CASEID; when both CASEID and FDA_DT were identical, the record with the largest PRIMARYID was retained. The PT analyzed of this study was cachexia, and its MedDRA Code was 10006895. We extracted all cases related to cachexia from the REAC files and restricted them to reports where the drug was regarded as the primary suspect drug (PS). Subsequently, we combined the REAC files with the DEMO files to obtain information on cachexia cases. Then, we integrated the REAC files with the DRUG files to acquire drug records associated with cachexia. Finally, the drugs related to cachexia were selected and ranked (Zhang et al., 2025).

### 2.2 Disproportionality analysis

The drugs were classified according to the Anatomical Therapeutic Chemical (ATC) classification system (https://atcddd.fhi.no/atc_ddd_index/). The report odds ratio (ROR), proportion report ratio (PRR), Bayesian confidence propagation neural network (BCPNN), and multi-item gamma poisson shrinker (MGPS) were used to analyze the correlation between the top 50 drugs in terms of report numbers and cachexia. These four methods were based on the calculation of a 2 × 2 contingency table, and the specific calculation standards are detailed in Supplementary Table S1 (Zorych et al., 2013). It was confirmed as a positive signal, when meeting the standards of all four methods simultaneously. For the drugs identified as positive signals, we drew volcano plots to visually represent the intensity of the signals. The ROR model was considered the main model for identifying signals of ADEs. The larger the ROR value, the stronger the signal, indicating a stronger correlation between the drug and malignancy. P-adjust was the P-value after Fisher’s exact test and Bonferroni correction. The time to onset (TTO) was calculated as the interval between the ADE date (EVENT_DT) and drug initiation date (START_DT). The median, interquartile range (IQR), and the Weibull shape parameter (WSP) test were applied to calculate and analyze TTO. The WSP test was characterized by scale parameter (α) and shape parameter (β). When β < 1 and 95%CI < 1, it indicated that the ADEs incidence rate decreased over time (early failure). When β = 1 and the 95%CI includes 1, it indicated that the incidence rate of ADEs continuously changed over time (random failure). When β > 1 and the 95% CI > 1, it suggested that the incidence rate of ADEs increased over time (wear - out failure) (Chen et al., 2025; Huoshen et al., 2026).

## 3 Results

### 3.1 Baseline characteristics of cachexia

**FIGURE 1:**
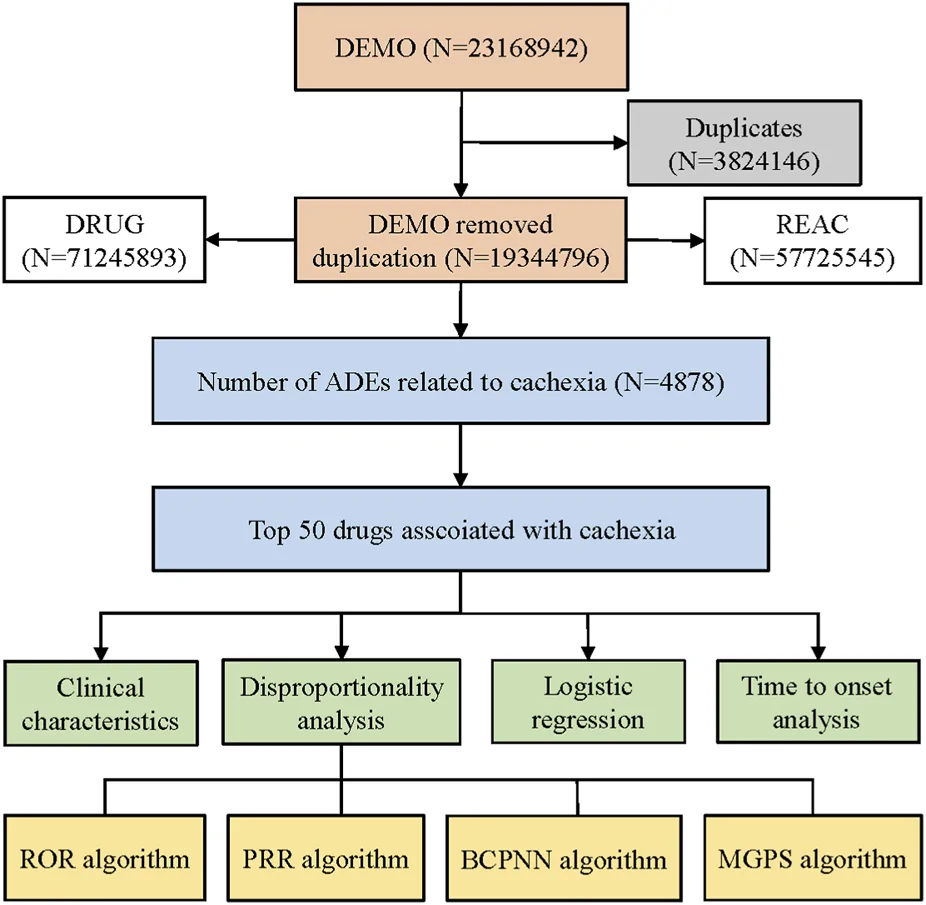
Flow chart for identifying ADEs related to cachexia. ADE, adverse drug event; DEMO, demographic and administrative details; REAC, reports of adverse events; DRUG, medication information; ROR, reporting odds ratio; PRR, proportional reporting ratio; BCPNN, Bayesian confidence propagation neural network; MGPS, multi-item gamma Poisson shrinker.

**FIGURE 2:**
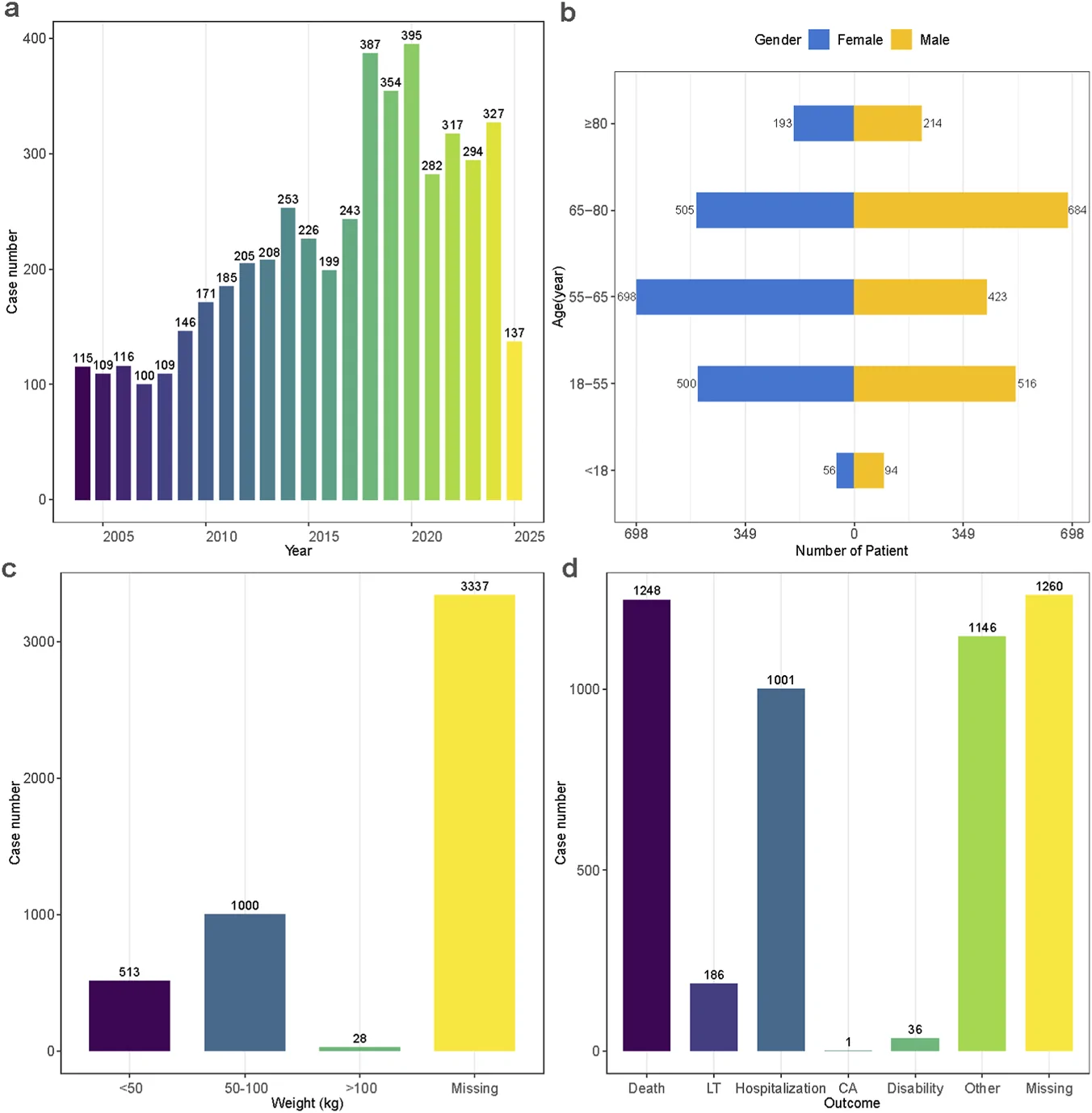
Baseline data from drug-related cachexia reports. **(a)** Drug-related cachexia reports by year; **(b)** Reporting rate of drug-related cachexia by patient age; **(c)** Reporting rate of drug-related cachexia by patient weight; **(d)** Adverse reactions’ outcome with drug-related cachexia.

The entire research process is shown in Figure 1. From the January 2004 and March 2025, the FAERS database recorded a total of 4,878 cases of cachexia. The baseline characteristics of these patients are presented in Table 1. Among them, there were 2,054 (42.1%) females and 2,427 (49.8%) males. The median age of the patients was 63 years (IQR 49–73 years), and the median weight was 56 kg (IQR 47–68 kg). 72.7% (3,548 cases) were reported by medical professionals. The top five countries in terms of the number of reports were the United States (1,082 cases), Germany (624 cases), France (413 cases), Japan (259 cases) and Canada (242 cases). The number of occurrences showed an overall upward trend year by year, with a higher number of occurrences from 2018 to 2020 (Figure 2a). The age of the overall population was concentrated in 18–80 years old. Among them, most female patients were 55–65 years old, while the majority of male patients are 65–80 years old (Figure 2b). The weight data of most patients was missing, and the weight was mostly between 50 and 100 kg among the available data (Figure 2c). Regarding the clinical outcome, the incidence of death among these patients was as high as 25.6% (Figure 2d).

**TABLE 1:** Clinical Characteristics of Patients with drug-related cachexia.

Characteristics	Drug-related cachexia (N = 4,878)
Gender	
Female	2054 (42.1%)
Male	2,427 (49.8%)
Unknown	397 (8.1%)
Age	
Median (Q1, Q3)	63.0 (49.0, 73.0)
Unknown	1,278 (26.2%)
Weight	
Median (Q1, Q3)	56.0 (47.0, 68.0)
Unknown	3,337 (68.4%)
Occupation of the reporter	
Physician	2068 (42.4%)
Pharmacist	223 (4.6%)
Other health-professional	1,257 (15.9%)
Lawyer	28 (0.6%)
Consumer	1,016 (25.8%)
Unknown	286 (5.9%)
Country of the reporter	
United States	1,082 (22.2%)
Germany	624 (12.8%)
France	413 (8.5%)
Japan	259 (5.3%)
Canada	242 (5.0%)
Other	2,258 (46.3%)

### 3.2 Drugs associated with cachexia

**FIGURE 3:**
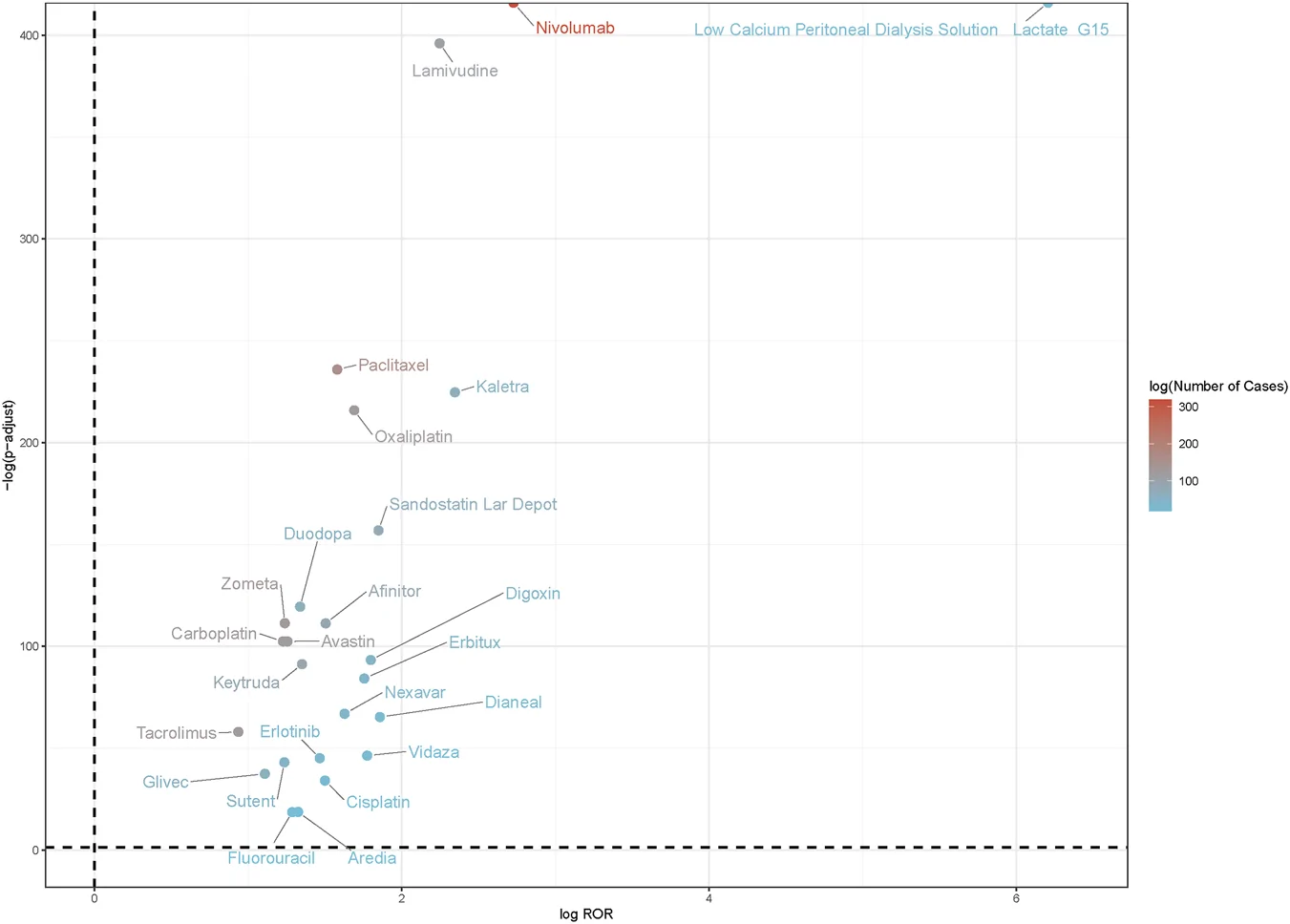
Volcano plots for the drugs identified as positive signals by the four models. ROR, reporting odds ratio; P-adjust, P-value after Bonferroni correction.

We conducted a disproportionality analysis of the top 50 drugs related to cachexia using the four models. These drugs were classified into 16 categories based on ATC classification system. A total of eight categories containing 25 drugs were identified as positive indicators related to cachexia (Table 2). Antineoplastic and immunomodulating agents (sixteen kinds of drugs) were the most common type of drugs related to cachexia, followed by blood and blood forming organs (two kinds of drugs), musculo-skeletal system (two kinds of drugs), antiinfectives for systemic use (two kinds of drug), nervous system (one kind of drugs), systemic hormonal preparations (one kind of drug), and cardiovascular system (one kind of drug). It is worth noting that none of the instructions of 25 drugs mentioned any ADEs related to cachexia. Instead, they merely mentioned digestive tract reactions such as nausea, vomiting, abdominal distension, and diarrhea, as well as metabolic disorders such as loss of appetite and electrolyte imbalance. A volcano plot was generated based on the ROR values and the number of cases (Figure 3). The x-axis displays the logarithm of ROR, and the positive x-axis indicates higher frequency of drug-related cachexia compared to other ADEs. The y-axis shows the negative logarithm of p-values after Fisher’s exact test and Bonferroni correction, and the positive y-axis indicates statistically significant differences. The color of each dot represents the logarithm of the number of case reports. The redder the color, the higher the number of reports. The drugs with stronger signals were low calcium peritoneal dialysis solution lactate G15 (ROR 495.67, 95%CI 336.94–729.18), Kaletra (ROR 10.44, 95%CI 7.98–13.65), and nivolumab (ROR 15.29, 95%CI 13.65–17.13). The drugs with a higher number of reported cases were nivolumab (319 cases), paclitaxel (160 cases), and Zometa (131 cases).

**TABLE 2:** The top 50 drugs associated with cachexia based on the number of adverse drug events reported.

Category	Drug	N	ROR (95%CI)	PRR (χ^2^)	EBGM (EBGM05)	IC (IC025)
Antineoplastic and immunomodulating agents	Nivolumab*	319	15.29 (13.65–17.13)	15.27 (3,976.87)	14.34 (12.80)	3.84 (3.62)
	Humira	94	0.57 (0.47–0.70)	0.57 (29.12)	0.58 (0.47)	−0.78 (−1.07)
	Avastin*	116	3.51 (2.92–4.22)	3.51 (203.20)	3.45 (2.87)	1.79 (1.49)
	Mycophenolate mofetil	151	2.37 (2.02–2.79)	2.37 (116.14)	2.33 (1.98)	1.22 (0.97)
	Remicade	53	0.82 (0.62–1.07)	0.82 (2.16)	0.82 (0.62)	−0.29 (−0.68)
	Rituximab	48	1.21 (0.91–1.60)	1.21 (1.66)	1.20 (0.91)	0.27 (−0.15)
	Glivec*	54	3.03 (2.31–3.96)	3.03 (72.43)	3.00 (2.30)	1.59 (1.14)
	Carboplatin*	122	3.41 (2.85–4.09)	3.41 (202.92)	3.35 (2.80)	1.75 (1.45)
	Oxaliplatin*	123	5.42 (4.54–6.49)	5.42 (432.48)	5.31 (4.44)	2.41 (2.10)
	Methotrexate	47	1.48 (1.11–1.98)	1.48 (7.36)	1.48 (1.11)	0.57 (0.13)
	Afinitor*	83	4.50 (3.62–5.59)	4.50 (222.01)	4.44 (3.57)	2.15 (1.77)
	Tacrolimus*	123	2.55 (2.13–3.05)	2.55 (113.08)	2.51 (2.10)	1.33 (1.05)
	Keytruda*	87	3.86 (3.12–4.77)	3.86 (180.88)	3.81 (3.08)	1.93 (1.57)
	Revlimid	42	0.66 (0.49–0.90)	0.66 (7.11)	0.67 (0.49)	−0.59 (−1.02)
	Nexavar*	41	5.09 (3.74–6.92)	5.09 (133.45)	5.05 (3.71)	2.34 (1.76)
	Paclitaxel*	160	4.85 (4.14–5.68)	4.85 (472.61)	4.72 (4.03)	2.24 (1.97)
	Erlotinib*	35	4.33 (3.11–6.04)	4.33 (89.10)	4.31 (3.09)	2.11 (1.50)
	Sutent*	49	3.44 (2.60–4.56)	3.44 (84.12)	3.42 (2.58)	1.77 (1.29)
	Capecitabine	32	2.30 (1.62–3.25)	2.30 (23.27)	2.29 (1.62)	1.19 (0.63)
	Erbitux*	43	5.79 (4.28–7.81)	5.78 (168.62)	5.74 (4.25)	2.52 (1.94)
	Cyclosporine	31	2.07 (1.46–2.95)	2.07 (17.10)	2.07 (1.45)	1.05 (0.49)
	Cisplatin*	25	4.48 (3.02–6.64)	4.48 (67.24)	4.46 (3.01)	2.16 (1.41)
	Enbrel	24	0.20 (0.13–0.30)	0.20 (77.13)	0.20 (0.14)	−2.30 (−2.83)
	Vidaza*	23	5.90 (3.92–8.89)	5.9 (93.20)	5.88 (3.90)	2.56 (1.70)
	Vedolizumab	22	1.05 (0.69–1.60)	1.05 (0.06)	1.05 (0.69)	0.07 (−0.53)
	Eligard	19	2.17 (1.38–3.40)	2.17 (11.87)	2.16 (1.38)	1.11 (0.38)
	Fluorouracil*	19	3.62 (2.31–5.68)	3.62 (35.89)	3.61 (2.30)	1.85 (1.03)
	Letrozole	17	2.43 (1.51–3.92)	2.43 (14.28)	2.43 (1.51)	1.28 (0.49)
	Pegasys	16	1.87 (1.15–3.06)	1.87 (6.46)	1.87 (1.14)	0.90 (0.13)
	Pazopanib	16	2.67 (1.64–4.37)	2.67 (16.70)	2.67 (1.63)	1.42 (0.58)
Nervous system	Duodopa*	57	6.11 (4.70–7.93)	6.10 (240.33)	6.04 (4.65)	2.60 (2.09)
	Clozaril	36	1.57 (1.13–2.18)	1.57 (7.39)	1.57 (1.13)	0.65 (0.15)
	Lyrica	27	0.83 (0.57–1.21)	0.83 (0.97)	0.83 (0.57)	−0.27 (−0.81)
Musculo-skeletal system	Zometa*	131	3.45 (2.90–4.10)	3.45 (221.36)	3.38 (2.84)	1.76 (1.48)
	Fosamax	23	0.97 (0.64–1.46)	0.97 (0.02)	0.97 (0.64)	−0.05 (−0.64)
	Ibuprofen	19	1.09 (0.70–1.72)	1.09 (0.15)	1.09 (0.70)	0.13 (−0.53)
	Aredia*	18	3.76 (2.37–5.97)	3.76 (36.31)	3.75 (2.36)	1.91 (1.05)
Systemic hormonal preparations, excl. sex hormones and insulins	Sandostatin lar depot*	71	6.35 (5.03–8.03)	6.35 (315.53)	6.27 (4.96)	2.65 (2.20)
	Prednisolone	22	2.56 (1.68–3.89)	2.56 (20.73)	2.55 (1.68)	1.35 (0.65)
	Prednisone	17	1.59 (0.99–2.56)	1.59 (3.68)	1.59 (0.98)	0.66 (−0.06)
Blood and blood forming organs	Clopidogrel	116	1.67 (1.39–2.01)	1.67 (30.64)	1.66 (1.38)	0.73 (0.45)
	Low calcium peritoneal dialysis solution lactate G15*	27	495.67 (336.94–729.18)	475.89 (12,725.23)	473.26 (321.70)	8.89 (4.17)
	Dianeal*	29	6.41 (4.45–9.23)	6.4 (131.43)	6.37 (4.42)	2.67 (1.90)
Alimentary tract and metabolism	Metformin	25	2.06 (1.39–3.05)	2.06 (13.5)	2.05 (1.38)	1.04 (0.41)
	Januvia	19	2.82 (1.80–4.43)	2.82 (22.26)	2.81 (1.79)	1.49 (0.72)
	Pantoprazole	17	2.37 (1.47–3.82)	2.37 (13.48)	2.37 (1.47)	1.24 (0.46)
Antiinfectives for systemic use	Kaletra*	54	10.44 (7.98–13.65)	10.43 (455.38)	10.33 (7.90)	3.37 (2.75)
	Lamivudine*	108	9.45 (7.81–11.44)	9.44 (797.44)	9.26 (7.65)	3.21 (2.83)
Cardiovascular system	Digoxin*	45	6.04 (4.50–8.10)	6.03 (187.22)	5.99 (4.46)	2.58 (2.01)
Various	Exjade	17	2.65 (1.64–4.26)	2.65 (17.37)	2.64 (1.64)	1.40 (0.59)

The asterisk (*) means statistically significant association with cachexia. ROR, reporting odds ratio; CI, confidence interval; PRR, proportional reporting ratio; BCPNN, Bayesian confidence propagation neural network; IC, information components; MGPS, multi-item gamma Poisson shrinker; EBGM, empirical Bayes geometric mean.

### 3.3 TTO analysis of cachexia

**FIGURE 4:**
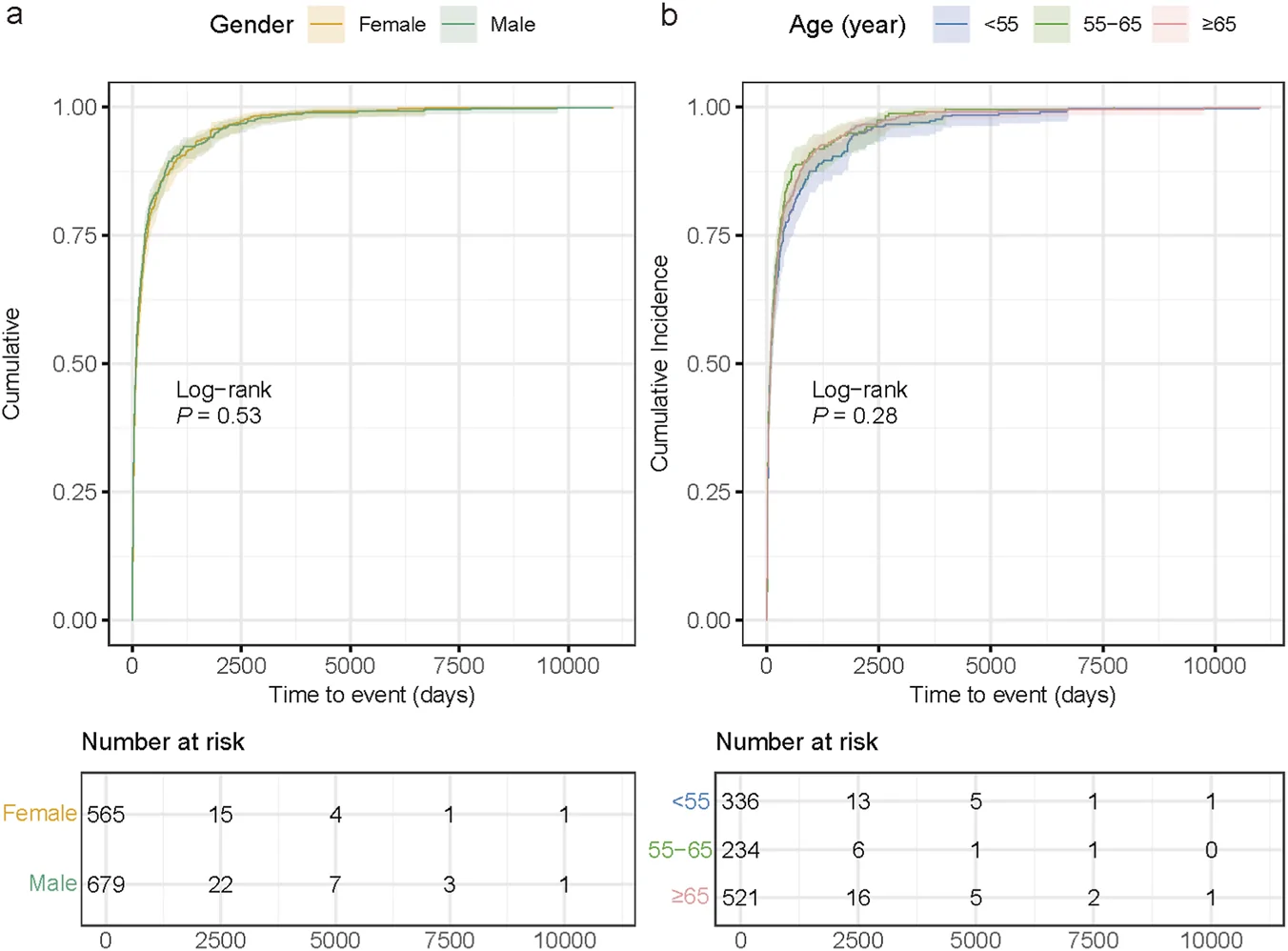
The time to onset of drug-related cachexia. **(a)** Cumulative incidence curve stratified by gender; **(b)** Cumulative incidence curve stratified by age group. P-values were calculated using the log-rank test.

1,310 reports contained complete TTO, and the median time of drug-related cachexia was 31 days (IQR 7–102 days), and approximately 57.3% of the reported cases occurred within the first 120 days after medication administration. Stratified analyses by gender and age revealed no significant differences in TTO distributions between males and females (Figure 4a), nor among patients aged <55 years, 55–65 years, and ≥65 years (Figure 4b).

The WSP analysis further characterized the temporal risk patterns across subgroups. For males and females, the shape parameter β were 0.57 (95%CI 0.53–0.60) and 0.54 (95%CI 0.51–0.57) respectively. The patients aged <55 years old, 55–65 years old and ≥65 years were 0.54 (95%CI 0.51–0.58), 0.56 (95%CI 0.51–0.61) and 0.54 (95%CI 0.51–0.58), respectively. Across all gender and age subgroups, the β values were consistently <1, indicating an early failure pattern in which the risk of cachexia decreases over time.

We performed drug-specific TTO analyses for drugs with ≥10 documented TTO reports to ensure robust parameter estimation. A total of 20 drugs with ≥10 documented TTO reports were included in the drug-specific TTO analysis. The median TTO varied substantially across drugs, ranging from 12.0 days (cisplatin) to 911.5 days (Duodopa). WSP analysis revealed that the majority of drugs (14/20, 70%) exhibited an early failure pattern, suggesting that the risk of cachexia is highest shortly after drug initiation. The remaining six drugs showed a random pattern, and no drug exhibited a wear-out pattern (Supplementary Table S2).

## 4 Discussion

This study represents the first large-scale pharmacovigilance analysis to systematically investigate and identify drugs associated with cachexia using the FAERS database. Our research has revealed an important yet often overlooked clinical issue: Among the 4,878 case safety reports, cachexia was identified as a potential ADE. What is particularly worrying is that the mortality rate of patients reporting drug-related cachexia reached 25.6%, highlighting the severity of this condition and its adverse prognosis—caused by underlying diseases, drug effects, or a combination of both. This mortality rate is substantially higher than that observed in many other drug-related ADEs, highlighting the devastating impact of cachexia on patient survival (von Haehling and Anker, 2014). The high mortality also suggests that drug-related cachexia might represent a more severe or rapidly progressive form of the syndrome, possibly due to the additive catabolic effects of the underlying disease and the pharmacological agent.

Our disproportionality analysis of the top 50 drugs most frequently reported with cachexia reveals a remarkably diverse pharmacological landscape associated with this debilitating syndrome. The identified drugs span nine distinct categories, underscoring that cachexia is not confined to a single drug class or therapeutic area but rather constitutes a cross-cutting ADE of modern pharmacotherapy. Notably, 25 of these 50 drugs generated positive safety signals across all four algorithms, indicating a robust statistical association. The antineoplastic and immunomodulating agents were the most prominently represented, which is in line with the known pathophysiological mechanisms of cancer cachexia. Beyond oncology, the significant signals for drugs used in HIV (Kaletra, lamivudine), Parkinson’s disease (Duodopa, levodopa/carbidopa), end-stage renal disease (peritoneal dialysis solutions), and other chronic conditions challenge the traditional view of cachexia as solely a complication of advanced malignancy or end-organ failure. It suggests a paradigm where the pharmacological intervention itself can be a primary or contributory driver of the catabolic state. The common threads linking many of these disparate agents appear to be their potential to induce or exacerbate chronic inflammation (e.g., ICIs, certain dialysis solutions), cause significant gastrointestinal toxicity (e.g., chemotherapeutics, mycophenolate), disrupt central metabolic pathways (e.g., mTOR inhibitors, somatostatin analogs), or induce mitochondrial dysfunction (e.g., older antiretrovirals). This indicates that when patients have underlying diseases, we should be more vigilant about cachexia.

Antineoplastic and immunomodulating agents constituted the largest category of drugs associated with cachexia reports. Among them, nivolumab has the highest number of reports and a very strong signal. Immunotherapy activates the systemic immune response in accordance with the chronic inflammatory driving characteristics of cancer cachexia. Elevated pro-inflammatory cytokines, such as IL-6 and TNF-α, and interferon-gamma (Groarke et al., 2024), can act through activating the ubiquitin-proteasome and autophagy-lysosome pathways, becoming potent inducers of muscle catabolism, while inhibiting anabolic signaling and appetite (Yeom and Yu, 2022). Immune-related ADEs such as colitis and hepatitis can also further contribute to cachexia by causing malabsorption, nutrient loss, and increasing metabolic demand. The high reporting frequency for ICIs may reflect their widespread use in advanced cancers that are themselves highly cachexigenic, but the strength of the disproportionality signal suggests a specific drug effect that potentially accelerates or unmasks the cachexia process in susceptible individuals. EGFR inhibitors, such as cetuximab and erlotinib, often cause severe skin toxicity (such as papulovesicular eruptions) and gastrointestinal reactions (diarrhea, mucositis), which affect nutrient intake and increase the metabolic demands for repair, leading to a net catabolic state (Lacouture, 2006). Everolimus not only has common side effects such as oral ulcers, rashes and fatigue, but also can inhibit the mTOR pathway, directly blocking this crucial anabolic pathway in the muscles (Sandri, 2016). Multi-kinase Inhibitors (sorafenib, sunitinib) are associated with a high incidence of diarrhea, hand-foot skin reaction, fatigue, and taste alteration. It is worth noting that research has shown that sorafenib can actively remodel skeletal muscle cells and induce cachexia by interfering with the epigenetic regulation and transcriptional signaling of muscle-specific genes (Khan et al., 2024). Platinum-based drugs such as oxaliplatin, cisplatin, and carboplatin are well-known for their high emetogenicity and neurotoxicity. They can also cause nephrotoxicity and electrolyte imbalance, further exacerbating the disruption of metabolic homeostasis. The identification of a broad spectrum of antineoplastic agents underscores the pervasive nature of cachexia as an adverse effect of cancer treatment. The mechanisms are often class-specific or even drug-specific. Cachexia is a recognized complication post-transplant, often termed “transplant cachexia.” The mechanisms are multifactorial: the underlying chronic illness necessitating transplantation, the catabolic stress of major surgery, and the immunosuppressive regimen itself (Aller de la Fuente, 2022). Furthermore, chronic immunosuppression predisposes to infections, which can trigger inflammatory cascades that accelerate cachexia.

Low calcium peritoneal dialysis solution lactate G15, as a type of Dianeal, demonstrated the most robust association with cachexia reports in our analysis. Peritoneal dialysis solutions, particularly those with specific compositions, are used in patients with end-stage renal disease, a population already predisposed to a condition known as protein-energy wasting or uremic cachexia (Carrero et al., 2013). The mechanism is multifactorial, involving chronic inflammation, metabolic acidosis, hormonal imbalances (e.g., resistance to insulin and growth hormone), and nutrient losses through the dialysate (Silva et al., 2021). The specific formulation of low calcium peritoneal dialysis solution lactate G15 might exacerbate this pre-existing risk. Lactate-buffered solutions have been implicated in inducing a pro-inflammatory state and potentially altering mesothelial cell biology, which could theoretically worsen the systemic inflammatory milieu that drives cachexia (Dervisoglu et al., 2008). Furthermore, the dialysis procedure itself can lead to significant protein loss into the dialysate, and the specific composition of this solution might inadequately correct metabolic acidosis or other uremic abnormalities, thereby accelerating muscle catabolism. Our finding suggests a potential drug-specific effect beyond the underlying renal disease, warranting further clinical investigation into the impact of different peritoneal dialysis solution compositions on cachexia progression.

The strong signal for Kaletra is consistent with the well-documented phenomenon of HIV-associated wasting syndrome, which shares many features with cancer cachexia (Keithley and Swanson, 2013). The mechanisms are multifactorial. Firstly, older protease inhibitors like lopinavir/ritonavir are associated with mitochondrial toxicity, which can impair cellular energy production and promote muscle apoptosis (Margolis et al., 2014). Secondly, they can cause significant gastrointestinal adverse effects (diarrhea, malabsorption) and taste disturbances, leading to reduced nutrient intake. Thirdly, chronic HIV infection itself drives persistent immune activation and inflammation, characterized by elevated levels of TNF-α, IL-1, and IL-6, which are central mediators of muscle catabolism (Giovanelli and Quinton, 2022). Kaletra may exacerbate this inflammatory state. Additionally, ritonavir, used as a pharmacokinetic booster, is a potent cytochrome P450 inhibitor and can cause metabolic complications like dyslipidemia and insulin resistance, creating an unfavorable metabolic environment for muscle maintenance (Calza et al., 2016). Lamivudine can cause mitochondrial toxicity due to inhibition of mitochondrial DNA polymerase-γ, leading to impaired oxidative phosphorylation, increased reactive oxygen species, and ultimately, cellular apoptosis (Brinkman et al., 1999). This is particularly detrimental to tissues with high energy demands, such as skeletal muscle. Mitochondrial dysfunction is a recognized early event in the development of muscle wasting in various cachexia models (Brown et al., 2017). Additionally, nucleoside reverse transcriptase inhibitors can cause lactic acidosis and hepatic steatosis, further disrupting metabolic homeostasis. The association of lamivudine with cachexia likely reflects its contribution to the mitochondrial toxicity component of HIV-associated wasting, even in virologically suppressed patients.

The significant association of these Parkinson’s disease medications with cachexia is intriguing. While Parkinson’s disease itself can lead to weight loss and malnutrition due to dysphagia, nausea, and increased energy expenditure from tremor and rigidity, the strong disproportionality signals suggest a pharmacological contribution (Sandri, 2016). Levodopa is a precursor to dopamine, and dopamine signaling plays a complex role in energy balance and reward pathways. Chronic dopaminergic therapy can lead to dopamine dysregulation syndrome, which may include appetite changes. Furthermore, a common side effect of levodopa is gastrointestinal distress, including nausea, vomiting, and anorexia, which can directly reduce caloric intake (Giovanelli and Quinton, 2022). The mechanism for Duodopa, a gel formulation for continuous intestinal infusion, might be related to more consistent drug exposure or local gastrointestinal effects of the continuous administration. It is also possible that the advanced Parkinson’s disease patients requiring such therapies are already in a vulnerable, pre-cachectic state due to their chronic neurodegenerative condition, and the medication exacerbates this predisposition.

Bisphosphonates are known to cause an acute-phase reaction after infusion, characterized by fever, myalgia, and flu-like symptoms, mediated by pro-inflammatory cytokines such as IL-6 and TNF-α (Drake et al., 2008). This transient inflammatory burst could theoretically contribute to a catabolic state. More importantly, a common side effect is gastrointestinal irritation, including nausea, dyspepsia, and abdominal pain. In the context of cancer patients, who are already at high risk for cachexia, these adverse effects could tip the balance towards significant weight loss. Furthermore, there is some evidence that nitrogen-containing bisphosphonates can indirectly affect muscle function, though the direct link to cachexia requires further exploration (Bone et al., 2017).

Octreotide inhibits the secretion of multiple hormones, including growth hormone, insulin, glucagon, and gastrin. Its link to cachexia can be explained by several mechanisms. By inhibiting growth hormone and insulin-like growth factor-1 (IGF-1), it suppresses key anabolic pathways crucial for muscle maintenance (Boguslawska and Korbonits, 2021). It also inhibits insulin secretion, which can promote a catabolic state and impair glucose utilization. Furthermore, octreotide causes gastrointestinal hypomotility, leading to bloating, discomfort, and early satiety, thereby reducing food intake. In patients with carcinoid syndrome, controlling hormone-related symptoms may improve quality of life, but the anti-anabolic effects of long-acting octreotide may simultaneously promote a slow, insidious form of cachexia, which our study highlights.

The main uses of cardiotonic drugs are to treat heart failure and atrial fibrillation. These two diseases are closely related to cardiac cachexia. It should be noted that the disproportionate analysis suggests that they may have drug-specific effects beyond the basic diseases (von Haehling et al., 2009). Digoxin can cause anorexia, nausea, and vomiting, which are classic drivers of reduced intake. More speculatively, digoxin’s mechanism involves inhibition of the Na^+^/K^+^-ATPase pump, which is fundamental to cellular electrolyte homeostasis. Chronic disruption of this pump in muscle cells could theoretically impair their viability and function, potentially contributing to muscle wasting. Additionally, digoxin has narrow therapeutic index, and its toxicity can manifest as severe gastrointestinal and neurological symptoms, potentially accelerating weight loss in vulnerable patients. Our findings suggest that in patients with heart failure who are developing cachexia, the role of digoxin should be critically evaluated.

Drugs such as rituximab, methotrexate, vedolizumab, and infliximab did not yield consistent positive signals for cachexia across all four disproportionality algorithms in our study. This finding is critical as it distinguishes drugs with robust potential associations with cachexia from those where the reporting frequency of cachexia is not disproportionately elevated relative to their overall ADE reporting profile. However, we caution against overinterpreting these negative findings. The absence of positive signals may be constrained by limited reporting numbers, low usage frequency, or reporting bias, and should not be interpreted as definitive evidence of no causal relationship with cachexia. FAERS data are intended for signal generation, not confirmatory analysis. For certain agents, particularly anti-TNF-α inhibitors like infliximab and etanercept, a theoretical association with cachexia might be anticipated given their anti-inflammatory properties; however, their primary application in autoimmune diseases (e.g., rheumatoid arthritis) that are themselves associated with cachexia risk complicates signal interpretation. The absence of positive signals for these drugs suggests that cachexia in such patients is more likely driven by the underlying autoimmune disease process—characterized by chronic systemic inflammation that activates catabolic pathways—rather than by the pharmacological effects of the drugs themselves.

The TTO analysis provided crucial insights into the temporal dynamics of drug-related cachexia. The median TTO was 31 days (IQR 7–102 days), with >50% of cases occurring within the first 120 days. Substantial heterogeneity was observed across individual drugs. Cisplatin showed the shortest median TTO (12.0 days), followed by Nexavar (28.0 days) and carboplatin (25.0 days), whereas Duodopa exhibited the longest (911.5 days), with tacrolimus (303.0 days) and Glivec (285.0 days) also showing markedly prolonged onset times. The Weibull shape parameter β indicated an “early failure” pattern across all demographic subgroups, meaning the highest risk occurs shortly after drug initiation and declines over time. Notably, fourteen drugs exhibited this early failure pattern. In contrast, six drugs (cisplatin, Sutent, Erbitux, Avastin, Keytruda, and Zometa) showed a random failure pattern, indicating constant hazard over time without an early peak. No drug showed a wear-out pattern, implying that prolonged exposure does not increase cachexia risk in our dataset.

The rapid onset observed in the majority of drugs likely reflects two interconnected mechanisms. First, acute drug toxicities—especially severe gastrointestinal effects (nausea, vomiting, diarrhoea, mucositis)—directly impair nutritional intake and absorption within days to weeks. This is particularly relevant for platinum-based chemotherapeutics and multi-kinase inhibitors (Bozzetti, 2020). ICIs such as nivolumab may trigger early immune-related colitis and hepatitis, further accelerating catabolism (Masuda et al., 2019). Second, many patients already harbour a pre-cachectic or early cachectic state from underlying chronic diseases (e.g., advanced cancer, heart failure, HIV, renal failure). The pharmacological insult may act as a “second hit” that tips the balance toward overt cachexia. This is supported by the high baseline prevalence of cachexia—up to 80% in advanced cancer (Koh et al., 2025) and 20%–30% in chronic heart failure (von Haehling et al., 2009). Clinically, these findings underscore the importance of intensified monitoring of weight, appetite, and nutritional status during the first 4 months—the highest-risk window—with proactive nutritional support (dietary counselling, oral supplements, appetite stimulants, or enteral support) to prevent refractory cachexia (Ahmad et al., 2022). Patients with pre-existing risk factors (advanced disease, low baseline weight, elevated inflammatory markers) may benefit from even earlier surveillance. The marked TTO variability across drugs further highlights the need to tailor monitoring intensity to the specific agent—e.g., more intensive early monitoring for cisplatin and nivolumab than for Duodopa and tacrolimus. Distinguishing *de novo* drug-induced cachexia from drug-exacerbated disease-related cachexia remains challenging, but the robust disproportionality signals combined with the early temporal pattern strongly suggest a causal or contributory role. Future prospective studies with baseline nutritional assessments, biomarkers, and body composition measurements are needed to unravel these mechanisms and identify highest-risk patients.

Several limitations inherent to this study must be acknowledged. First, as a spontaneous reporting system, FAERS is susceptible to under-reporting, reporting biases, and variable data quality and completeness. In particular, reports submitted by non-healthcare professionals carry a higher risk of diagnostic inaccuracy or misclassification of cachexia, as recognition of this syndrome requires clinical expertise. Additionally, the FAERS database is heavily weighted toward reports from the United States and a few other countries, introducing a geographical reporting bias that may limit the generalisability of our findings to other regions. Second, the absence of a control population and detailed clinical information prevents the calculation of true incidence and limits the ability to control for all potential confounders. Third, we cannot definitively distinguish between true drug-induced cachexia and drug-exacerbated cachexia in a patient whose wasting was primarily driven by their underlying disease (e.g., cancer, HIV, heart failure). The reported association could mean the drug triggered cachexia *de novo*, or more likely, that it significantly worsened an existing condition. Fourth, our case definition relied on a single Preferred Term (PT) “cachexia” rather than a broader set of terms. We deliberately adopted this focused definition to maintain clinical specificity, as cachexia is pathophysiologically distinct from related conditions such as sarcopenia or isolated muscle atrophy—the former involves systemic inflammation and involuntary loss of both muscle and fat mass, whereas the latter primarily refers to age-related muscle loss. However, we acknowledge that this approach may have led to under-ascertainment of cases. Finally, the identified signals indicate statistical associations, not proven causation, and require validation through prospective, controlled clinical studies and mechanistic investigations.

## 5 Conclusion

This study provides a comprehensive “risk map” of pharmaceuticals associated with cachexia across diverse therapeutic areas. We identified 25 drugs with positive signals from the 50 drugs most frequently associated with cachexia. The risk of developing cachexia is highest in the early stage of administration, especially during the first 4 months of treatment; therefore, it is necessary to actively monitor cachexia symptoms in patients receiving these medications. The high mortality associated with these reports should elevate the concern for this adverse effect. Furthermore, our list of 25 drugs, none of which currently mention cachexia in their labels, provides a strong evidence base for regulatory bodies to consider updating drug safety information. Future research should focus on validating these associations in specific patient cohorts and elucidating the underlying mechanisms to develop targeted interventions that can prevent or treat this devastating complication of drug therapy.

## Data Availability

The original contributions presented in the study are included in the article/Supplementary Material, further inquiries can be directed to the corresponding authors.

## References

[B1] Ahmad S. S. Ahmad K. Shaikh S. You H. J. Lee E. Y. Ali S. (2022). Molecular mechanisms and current treatment options for cancer cachexia. Cancers (Basel) 14, 2107. 10.3390/cancers14092107 35565236 PMC9105812

[B2] Aller de la Fuente R. (2022). Nutrition and chronic liver disease. Clin. Drug Investig. 42, 55–61. 10.1007/s40261-022-01141-x 35484325 PMC9205793

[B3] Argiles J. M. Busquets S. Stemmler B. Lopez-Soriano F. J. (2014). Cancer cachexia: understanding the molecular basis. Nat. Rev. Cancer 14, 754–762. 10.1038/nrc3829 25291291

[B4] Boguslawska A. Korbonits M. (2021). Genetics of acromegaly and gigantism. J. Clin. Med. 10, 1377. 10.3390/jcm10071377 33805450 PMC8036715

[B5] Bone H. G. Wagman R. B. Brandi M. L. Brown J. P. Chapurlat R. Cummings S. R. (2017). 10 years of denosumab treatment in postmenopausal women with osteoporosis: results from the phase 3 randomised FREEDOM trial and open-label extension. Lancet Diabetes Endocrinol. 5, 513–523. 10.1016/S2213-8587(17)30138-9 28546097

[B6] Bozzetti F. (2020). Chemotherapy-induced sarcopenia. Curr. Treat. Options Oncol. 21, 7. 10.1007/s11864-019-0691-9 32002684

[B7] Brinkman K. Smeitink J. A. Romijn J. A. Reiss P. (1999). Mitochondrial toxicity induced by nucleoside-analogue reverse-transcriptase inhibitors is a key factor in the pathogenesis of antiretroviral-therapy-related lipodystrophy. Lancet 354, 1112–1115. 10.1016/S0140-6736(99)06102-4 10509516

[B8] Brown J. L. Rosa-Caldwell M. E. Lee D. E. Blackwell T. A. Brown L. A. Perry R. A. (2017). Mitochondrial degeneration precedes the development of muscle atrophy in progression of cancer cachexia in tumour-bearing mice. J. Cachexia Sarcopenia Muscle 8, 926–938. 10.1002/jcsm.12232 28845591 PMC5700433

[B9] Calza L. Colangeli V. Manfredi R. Bon I. Re M. C. Viale P. (2016). Clinical management of dyslipidaemia associated with combination antiretroviral therapy in HIV-Infected patients. J. Antimicrob. Chemother. 71, 1451–1465. 10.1093/jac/dkv494 26846208

[B10] Carrero J. J. Stenvinkel P. Cuppari L. Ikizler T. A. Kalantar-Zadeh K. Kaysen G. (2013). Etiology of the protein-energy wasting syndrome in chronic kidney disease: a consensus statement from the International Society of Renal Nutrition and Metabolism (ISRNM). J. Ren. Nutr. 23, 77–90. 10.1053/j.jrn.2013.01.001 23428357

[B11] Chen H. Liu S. Gao S. Shi H. Yan Y. Xu Y. (2025). Pharmacovigilance analysis of neurological adverse events associated with GLP-1 receptor agonists based on the FDA Adverse Event Reporting System. Sci. Rep. 15, 18063. 10.1038/s41598-025-01206-9 40413246 PMC12103604

[B12] Dervisoglu E. Eraldemir C. Kalender B. Kir H. M. Caglayan C. (2008). Adipocytokines leptin and adiponectin, and measures of malnutrition-inflammation in chronic renal failure: is there a relationship? J. Ren. Nutr. 18, 332–337. 10.1053/j.jrn.2008.02.001 18558297

[B13] Drake M. T. Clarke B. L. Khosla S. (2008). Bisphosphonates: mechanism of action and role in clinical practice. Mayo Clin. Proc. 83, 1032–1045. 10.4065/83.9.1032 18775204 PMC2667901

[B14] Giovanelli L. Quinton R. (2022). Therapeutic effects of androgens for cachexia. Best. Pract. Res. Clin. Endocrinol. Metab. 36, 101598. 10.1016/j.beem.2021.101598 34801415

[B15] Groarke J. D. Crawford J. Collins S. M. Lubaczewski S. Roeland E. J. Naito T. (2024). Ponsegromab for the treatment of cancer cachexia. N. Engl. J. Med. 391, 2291–2303. 10.1056/nejmoa2409515 39282907

[B16] Huoshen W. Xiong J. Ma X. Wang H. Cheng P. Chen X. (2026). Pharmacovigilance-based identification and mechanistic exploration of periodontitis-related drugs. J. Clin. Periodontol. 53, 117–126. 10.1111/jcpe.70040 40957584 PMC12695453

[B17] Kandukuru A. Sharma P. Verghese Gupta S. Nkembo A. Sutariya V. (2024). Cardiovascular adverse events associated with norepinephrine-dopamine reuptake inhibitors: a pharmacovigilance study of the FDA Adverse Event Reporting System. Can. J. Physiol. Pharmacol. 102, 709–719. 10.1139/cjpp-2024-0128 39431859

[B18] Keithley J. K. Swanson B. (2013). HIV-associated wasting. J. Assoc. Nurses AIDS Care 24, S103–S111. 10.1016/j.jana.2012.06.013 23290370

[B19] Khan B. Lanzuolo C. Rosti V. Santarelli P. Pich A. Kraft T. (2024). Sorafenib induces cachexia by impeding transcriptional signaling of the SET1/MLL complex on muscle-specific genes. iScience 27, 110913. 10.1016/j.isci.2024.110913 39386761 PMC11462028

[B20] Koh K. Scott R. Cespedes Feliciano E. M. Janowitz T. Goncalves M. D. White E. P. (2025). Cancer-associated cachexia: bridging clinical findings with mechanistic insights in human studies. Cancer Discov. 15, 1543–1568. 10.1158/2159-8290.CD-25-0293 40298389 PMC12794921

[B21] Lacouture M. E. (2006). Mechanisms of cutaneous toxicities to EGFR inhibitors. Nat. Rev. Cancer 6, 803–812. 10.1038/nrc1970 16990857

[B22] Lin K. Wei L. Wang R. Li L. Song S. Wang F. (2025). Disrupted methionine cycle triggers muscle atrophy in cancer cachexia through epigenetic regulation of REDD1. Cell Metab. 37, 460–476 e8. 10.1016/j.cmet.2024.10.017 39729999

[B23] Margolis A. M. Heverling H. Pham P. A. Stolbach A. (2014). A review of the toxicity of HIV medications. J. Med. Toxicol. 10, 26–39. 10.1007/s13181-013-0325-8 23963694 PMC3951641

[B24] Masuda K. Shoji H. Nagashima K. Yamamoto S. Ishikawa M. Imazeki H. (2019). Correlation between immune-related adverse events and prognosis in patients with gastric cancer treated with nivolumab. BMC Cancer 19, 974. 10.1186/s12885-019-6150-y 31638948 PMC6805586

[B25] Meregaglia M. Borsoi L. Cairns J. Tarricone R. (2019). Mapping health-related quality of life scores from FACT-G, FAACT, and FACIT-F onto preference-based EQ-5D-5L utilities in non-small cell lung cancer cachexia. Eur. J. Health Econ. 20, 181–193. 10.1007/s10198-017-0930-6 28948436 PMC6438942

[B26] Rohm M. Zeigerer A. Machado J. Herzig S. (2019). Energy metabolism in cachexia. EMBO Rep. 20, e47258. 10.15252/embr.201847258 30890538 PMC6446208

[B27] Sandri M. (2016). Protein breakdown in cancer cachexia. Semin. Cell Dev. Biol. 54, 11–19. 10.1016/j.semcdb.2015.11.002 26564688

[B28] Silva M. Z. C. Vogt B. P. Reis N. S. C. Oliveira R. C. Caramori J. C. T. (2021). Which method should be used to assess protein intake in peritoneal dialysis patients? Assessment of agreement between protein equivalent of total nitrogen appearance and 24-Hour dietary recall. J. Ren. Nutr. 31, 320–326. 10.1053/j.jrn.2020.08.003 32958375

[B29] Tan H. Chen X. Chen Y. Ou X. Yang T. Yan X. (2025). Immune checkpoint inhibitor-associated bullous pemphigoid: a retrospective and real-world study based on the United States Food and Drug Administration adverse event reporting system. J. Dermatol 52, 309–316. 10.1111/1346-8138.17517 39460482

[B30] Tarricone R. Ricca G. Nyanzi-Wakholi B. Medina-Lara A. (2016). Impact of cancer anorexia-cachexia syndrome on health-related quality of life and resource utilisation: a systematic review. Crit. Rev. Oncol. Hematol. 99, 49–62. 10.1016/j.critrevonc.2015.12.008 26775729

[B31] von Haehling S. Anker S. D. (2014). Prevalence, incidence and clinical impact of cachexia: facts and numbers-update 2014. J. Cachexia Sarcopenia Muscle 5, 261–263. 10.1007/s13539-014-0164-8 25384990 PMC4248411

[B32] von Haehling S. Lainscak M. Springer J. Anker S. D. (2009). Cardiac cachexia: a systematic overview. Pharmacol. Ther. 121, 227–252. 10.1016/j.pharmthera.2008.09.009 19061914

[B33] Yang X. Zhang X. Heckmann B. L. Lu X. Liu J. (2011). Relative contribution of adipose triglyceride lipase and hormone-sensitive lipase to tumor necrosis factor-alpha (TNF-alpha)-induced lipolysis in adipocytes. J. Biol. Chem. 286, 40477–40485. 10.1074/jbc.M111.257923 21969372 PMC3220500

[B34] Yao S. Yang Z. Li J. Peng B. Wang H. Liang J. (2025). Prevalence and prognostic significance of cachexia diagnosed by novel definition for Asian population among Chinese cirrhotic patients. Arch. Gerontol. Geriatr. 133, 105833. 10.1016/j.archger.2025.105833 40120202

[B35] Yeom E. Yu K. (2022). Understanding the molecular basis of anorexia and tissue wasting in cancer cachexia. Exp. Mol. Med. 54, 426–432. 10.1038/s12276-022-00752-w 35388147 PMC9076846

[B36] Zhang Y. Deng W. Wang M. Luo S. Li S. (2024). A real-world pharmacovigilance study of neuroleptic malignant syndrome based on FDA adverse event reporting system. Front. Pharmacol. 15, 1438661. 10.3389/fphar.2024.1438661 39723245 PMC11668602

[B37] Zhang X. Li X. Huoshen W. Li S. (2025). Identification of drugs associated with oral ulcers based on pharmacovigilance. Int. Dent. J. 75, 103957. 10.1016/j.identj.2025.103957 41092835 PMC12552536

[B38] Zhao B. Shi G. Shi J. Li Z. Xiao Y. Qiu Y. (2025). Research progress on the mechanism and treatment of cachexia based on tumor microenvironment. Nutrition 133, 112697. 10.1016/j.nut.2025.112697 39999652

[B39] Zorych I. Madigan D. Ryan P. Bate A. (2013). Disproportionality methods for pharmacovigilance in longitudinal observational databases. Stat. Methods Med. Res. 22, 39–56. 10.1177/0962280211403602 21878461

