# Supplementary material for: Risk factors for drug-related cachexia: an analysis based on the FDA adverse event reporting system (FAERS) from 2004 to 2025
Source: Front Pharmacol. 2026 Sep 8;17:1913539. doi: 10.3389/fphar.2026.1913539 (PMC13593894; doi:10.3389/fphar.2026.1913539)
Supplement: Supplementary file 1 [file Supplementaryfile1.docx]

Table S1 Four models used for signal detection

| Models | Equation | Criteria |
| --- | --- | --- |
| ROR | ROR=(a/b)/(c/d) | 95% CI lower>1, a≥3 |
| PRR | PRR=[a/(a+c)]/[b/(b+d)];  χ²=∑[(O-E)2/E];  (O=a, E=(a+b)(a+c)/(a+b+c+d) | PRR≥2, χ²≥4, a≥3 |
| BCPNN | IC=log_2_a(a+b+c+d)/[(a+c)(a+b)] | 95% CI lower (IC025)>0 |
| MGPS | EBGM=a(a+b+c+d)/[(a+c)(a+b)] | 95%CI (EBGM05)>2, a>0 |

ROR: reporting odds ratio; BCPNN: Bayesian confidence propagation neural network; CI: confidence interval; a: the number of reports containing both target drug and target adverse drug reaction; b: the number of reports containing the target adverse drug reaction with other medications (except the target drug); c: the number of reports containing the target drug with other adverse drug reactions (except the target events); d: the number of all reports.

Table S2 TTO and Weibull shape parameter of cachexia by drug category

| Drug | N | Median TTO (IQR), days | Weibull β (shape) | Hazard trend interpretation |
| --- | --- | --- | --- | --- |
| Cisplatin | 11 | 12.0 (8.5–18.0) | 1.24 (0.68–1.80) | Random |
| Nexavar | 39 | 18.0 (9.5–62.5) | 0.72 (0.50–0.93) | Early failure |
| Carboplatin | 38 | 25.0 (9.3–75.8) | 0.70 (0.53–0.87) | Early failure |
| Nivolumab | 59 | 28.0 (10.0–129.5) | 0.64 (0.51–0.76) | Early failure |
| Erlotinib | 16 | 31.0 (11.0–55.3) | 0.74 (0.59–0.89) | Early failure |
| Sutent | 18 | 32.0 (15.3–110.5) | 0.83 (0.53–1.13) | Random |
| Oxaliplatin | 45 | 34.0 (11.0–133.0) | 0.80 (0.62–0.98) | Early failure |
| Erbitux | 21 | 43.0 (24.0–88.0) | 1.03 (0.71–1.35) | Random |
| Paclitaxel | 11 | 48.5 (14.0–79.0) | 0.74 (0.59–0.89) | Early failure |
| Avastin | 43 | 66.0 (20.5–176.0) | 0.86 (0.66–1.05) | Random |
| Afinitor | 35 | 67.0 (19.0–150.0) | 0.65 (0.49–0.80) | Early failure |
| Keytruda | 14 | 68.5 (31.5–84.3) | 1.10 (0.64–1.57) | Random |
| Clopidogrel | 24 | 82.0 (42.3–330.5) | 0.53 (0.38–0.68) | Early failure |
| Lamivudine | 37 | 98.0 (40.0–378.0) | 0.72 (0.55–0.90) | Early failure |
| Kaletra | 35 | 108.0 (39.0–366.0) | 0.56 (0.43–0.69) | Early failure |
| Zometa | 46 | 139.0 (50.8–328.5) | 0.90 (0.70–1.11) | Random |
| Sandostatin Lar Depot | 24 | 200.0 (80.0–1367.5) | 0.66 (0.46–0.87) | Early failure |
| Glivec | 17 | 285.0 (35.0–730.0) | 0.64 (0.39–0.89) | Early failure |
| Tacrolimus | 10 | 303.0 (60.0–667.5) | 0.61 (0.30–0.91) | Early failure |
| Duodopa | 18 | 911.5 (186.8–1580.5) | 0.68 (0.41–0.96) | Early failure |

TTO: time-to-onset; IQR: interquartile range; CI: confidence interval.
